# Identification of Key Genes and Pathways in Myeloma side population cells by Bioinformatics Analysis

**DOI:** 10.7150/ijms.48244

**Published:** 2020-07-25

**Authors:** Qin Yang, Kaihu Li, Xin Li, Jing Liu

**Affiliations:** 1 Department of Hematology, the Third Xiangya Hospital, Central South University, Changsha, Hunan, P.R. China.; 2 Department of Orthopaedics, Xiangya Hospital, Central South University, Changsha, Hunan, P.R. China.

**Keywords:** Multiple myeloma, Cancer stem cell, Side population cells, Bioinformatics analysis, differentially expressed gene

## Abstract

**Background:** Multiple myeloma (MM) is the second most common hematological malignancy, which is still incurable and relapses inevitably, highlighting further understanding of the possible mechanisms. Side population (SP) cells are a group of enriched progenitor cells showing stem-like phenotypes with a distinct low-staining pattern with Hoechst 33342. Compared to main population (MP) cells, the underlying molecular characteristics of SP cells remain largely unclear. This bioinformatics analysis aimed to identify key genes and pathways in myeloma SP cells to provide novel biomarkers, predict MM prognosis and advance potential therapeutic targets.

**Methods:** The gene expression profile GSE109651 was obtained from Gene Expression Omnibus database, and then differentially expressed genes (DEGs) with P-value <0.05 and |log2 fold-change (FC)| > 2 were selected by the comparison of myeloma light-chain (LC) restricted SP (LC/SP) cells and MP CD138^+^ cells. Subsequently, gene ontology (GO) and Kyoto encyclopedia of genes and genomes (KEGG) pathway enrichment analysis, protein-protein interaction (PPI) network analysis were performed to identify the functional enrichment analysis of the DEGs and screen hub genes. Cox proportional hazards regression was used to select the potential prognostic DEGs in training dataset (GSE2658). The prognostic value of the potential prognostic genes was evaluated by Kaplan-Meier curve and validated in another external dataset (MMRF-CoMMpass cohort from TCGA).

**Results:** Altogether, 403 up-regulated and 393 down-regulated DEGs were identified. GO analysis showed that the up-regulated DEGs were significantly enriched in innate immune response, inflammatory response, plasma membrane and integral component of membrane, while the down-regulated DEGs were mainly involved in protoporphyrinogen IX and heme biosynthetic process, hemoglobin complex and erythrocyte differentiation. KEGG pathway analysis suggested that the DEGs were significantly enriched in osteoclast differentiation, porphyrin and chlorophyll metabolism and cytokine-cytokine receptor interaction. The top 10 hub genes, identified by the plug-in cytoHubba of the Cytoscape software using maximal clique centrality (MCC) algorithm, were ITGAM, MMP9, ITGB2, FPR2, C3AR1, CXCL1, CYBB, LILRB2, HP and FCER1G. Modules and corresponding GO enrichment analysis indicated that myeloma LC/SP cells were significantly associated with immune system, immune response and cell cycle. The predictive value of the prognostic model including TFF3, EPDR1, MACROD1, ARHGEF12, AMMECR1, NFATC2, HES6, PLEK2 and SNCA was identified, and validated in another external dataset (MMRF-CoMMpass cohort from TCGA).

**Conclusions:** In conclusion, this study provides reliable molecular biomarkers for screening, prognosis, as well as novel therapeutic targets for myeloma LC/SP cells.

## Introduction

Multiple myeloma (MM) is a B-cell malignancy characterized by the aberrant expansion of clonal plasma cells within bone marrow, which is the second most common hematological malignancy 1. Despite remarkable progress of biology and recent development of novel therapy 2, 3, MM continues to remain incurable due to the emergence of drug resistance and frequent relapses, highlighting the further understanding of the possible mechanisms.

Cancer stem cells (CSCs) are thought to have the distinctive properties of constituting a small fraction of tumor cells with self-renewal capacity and be able to propagate the disease 4, 5. Besides, CSCs are considered to be more resistant to chemo- and radio-therapy and have better DNA repair mechanisms and increased anti-apoptotic activity, just like hematopoietic stem cells 6. Previously, CSCs have been identified in MM 7 by the evidence that the CD138^-^/CD19^+^ fraction of MM has a greater clonogenic potential and the phenotype of a memory B-cell (CD19^+^, CD27^+^), resulting in the development of refractory clones and disease relapse^8^. Then, it has been defined that possible stem cell populations include light-chain restricted cells with a CD138^-^/CD19^+^/CD27^+^ phenotype 7, 9, 10, CD138^+^/CD34^+^/B7^-^H1^+^ subpopulations 11 and CD38^++^/CD45^-^ plasma cells 12, 13. Despite these phenotypes have been described, the distinct CSCs marker in MM is still controversial.

Side population (SP) cells, first described by Goodell et al. 14, are a group of enriched progenitor cells showing stem-like phenotypes and a distinct low-staining pattern with Hoechst 33342, and have been widely used as a unique source for studying CSCs in the absence of specific markers 4, 15-21. Although lots of previous studies have explored the stem-like properties and tumorigenicity of myeloma SP cells, a better understanding of SP cells still remains largely unclear 10, 22-25. Thus, it is vital to elucidate the key molecular characteristics expressed within myeloma SP cells.

It is generally known that gene expression profiling analysis based on microarray technology enables the possibilities for identifying certain disease-related biomarkers. Recently, many studies have been carried out on the base of microarray data profiles to identify the pathogenesis of MM 26-28. Nevertheless, the key molecular characteristics of myeloma SP cells in comparison to MP cells have not yet been explored. This bioinformatics analysis was performed to elucidate key candidate genes and pathways in myeloma SP cells, provide novel biomarkers, predict MM prognosis and advance potential therapeutic targets.

In this study, we downloaded microarray dataset GSE109651 (Zhan et al., 2018) from Gene Expression Omnibus database (https://www.ncbi.nlm.nih.gov/geo/), which is a public functional genomics data repository with array- and sequence-based data. By comparing myeloma light-chain (LC) restricted SP (LC/SP) cells with myeloma MP cells based on R software and Bioconductor, differentially expressed genes (DEGs) were identified. Gene Ontology (GO) analysis, Kyoto encyclopedia of genes and genomes (KEGG) pathway analysis and protein-protein interaction (PPI) network analysis were performed to identify the functional enrichment analysis of the DEGs and screen hub genes. Subsequently, we constructed a prognostic model to predict survivals of MM patients. This study provides reliable molecular biomarkers for screening, prognosis, as well as novel therapeutic targets for LC/SP cells of MM.

## Materials & Methods

### Microarray data profile

The gene expression dataset GSE109651 was obtained from GEO database. The microarray data of GSE109651, based on the GPL570 platform ([HG-U133_Plus_2] Affymetrix Human Genome U133A Plus 2.0 Array) and normalized using the MAS5 algorithm of the Affymetrix expression console version1.1 software (Affymetrix), includes 7-paired LC/SP cells and MP CD138^+^ cells of myeloma bone marrow from 7 diagnosed MM patients isolated by fluorescence-activated cell sorting (FACS) using Hoechst 33342 and CD138 antibody. To perform survival analysis, GSE2658 dataset of 559 MM patients and TCGA MM RNA sequencing dataset (MMRF-CoMMpass) of 787 cases with MM including clinicopathological information were downloaded from GEO database and TCGA (https://tcga-data.nci.nih.gov/) databases, respectively. For the retrospective cohort, the patients' characteristics were estimated by Pearson test χ^2^ or Fisher's exact test, indicating no significant statistical difference.

### Data processing and identification of DEGs of GSE109651

Firstly, we detected the quality of raw data by R statistical software (version 3.6.3, https://www.r-project.org/), including a quality control overview diagram based on the “simpleaffy” package, weights and residuals plot, relative log expression (RLE) boxplot and normalized unscaled standard errors (NUSE) box plot based on the "affyPLM" and "RColorBrewer" packages, RNA degradation curve based on the "affy" package and clustering analysis diagram based on the "gcrma", "graph" and "affycoretools" packages.

Then, DEGs between LC/SP cells and MP CD138^+^ cells of MM were identified by an empirical Bayes method based on the “limma” package in R. The process included six main steps: construction of a gene expression matrix, construction of an experimental design matrix, construction of a contrast matrix, fitting of a linear model, Bayes test, and generation of results. In this study, genes with P-value < 0.05 and |log2 fold-change (FC)| > 2 were defined as DEGs.

### GO and KEGG pathway enrichment analysis of DEGs

To explore the functional roles of the above DEGs, DAVID database (https://david.ncifcrf.gov/) was used to perform GO term enrichment analysis of molecular function (MF), biological process (BP), and cellular component (CC) and KEGG pathway enrichment analysis. P-value < 0.05 was considered as the cut-off criterion.

### PPI network construction and modular analysis

Search Tool for the Retrieval of Interacting Genes (STRING) database (http://www.string-db.org/) was used to construct the PPI network. The visualization and analysis of the PPI network were based on Cytoscape software version 3.7.2. Then, the plug-ins Molecular Complex Detection (MCODE) and Biological Network Gene Ontology tool (BiNGO) in Cytoscape software were used to screen significant modules of the PPI network (the parameters were set to default) and perform GO analysis that the module genes were significantly enriched in.

### Identification of hub genes

The plug-in cytoHubba in Cytoscape was used to identify key (hub) genes among the above DEGs by maximal clique centrality (MCC) computing method. The hub genes were selected to discuss their function and effect on myeloma LC/SP cells.

### Survival analysis

DEGs significantly associated with myeloma-specific survival in the training dataset (GSE2658) were identified using univariate Cox proportional hazards analysis with P-value < 0.01 by “survival” package 29. Then, the final genes significantly correlated with survival at a P-value of less than 0.05 were identified by multivariate Cox proportional hazards analysis. Subsequently, the risk score on the base of the aforementioned candidate genes and survival information was calculated as follows: Risk score = *∑β i × ExpGene i* (*β i* was the coefficient value and *ExpGene i* was the gene expression level). According to the median risk score, the cohort was dichotomized into low-risk and high-risk group, then survival time was compared by the Kaplan-Meier analysis and the log-rank test with a P-value of less than 0.01. Another external dataset (MMRF-CoMMpass cohort) was used to assess the prognostic value through a process similar to the training dataset.

## Results

### Identification of DEGs

**Figure 1:**
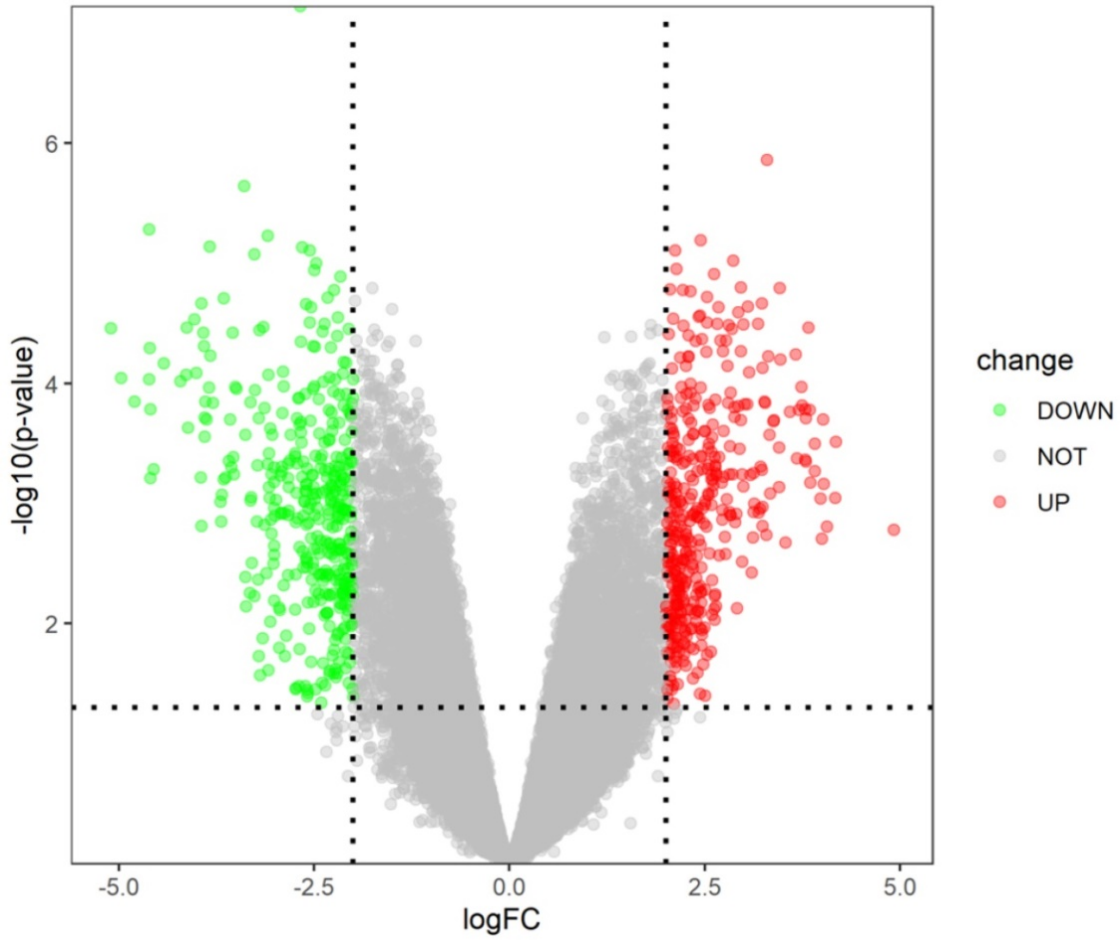
Volcano plot of DEGs (393 down-regulated genes and 403 up-regulated genes).

**Figure 2:**
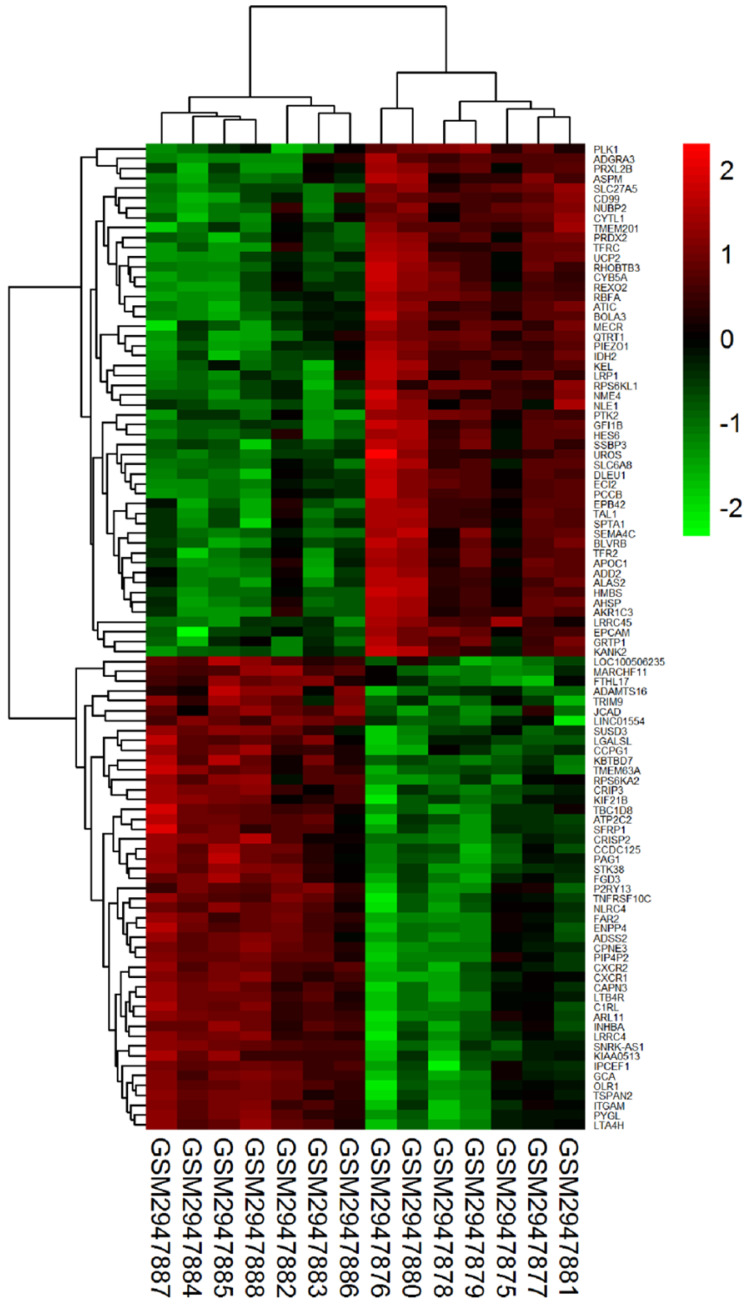
Heat map of the top 100 DEGs (52 down-regulated genes and 48 up-regulated genes).

The gene expression dataset GSE109651 included 7-paired LC/SP cells samples and MP CD138^+^ cells samples of myeloma bone marrow. On the basis of cut-off criterion of DEGs described previously, there were 796 DEGs in LC/SP cells compared with MP CD138^+^ cells of myeloma bone marrow, among which 393 DEGs were significantly down-regulated and 403 DGEs were significantly up-regulated. The volcano plot of DEGs was shown in Figure 1. The expression heat map of the top 100 DEGs (including 52 significantly down-regulated genes and 48 significantly up-regulated genes) was depicted in Figure 2, which could effectively distinguish LC/SP cells from MP CD138^+^ cells and might function as biomarker and target of MM. The detailed information of the top 10 DEGs was shown in Table 1.

### GO term enrichment analysis of DEGs

**Figure 3:**
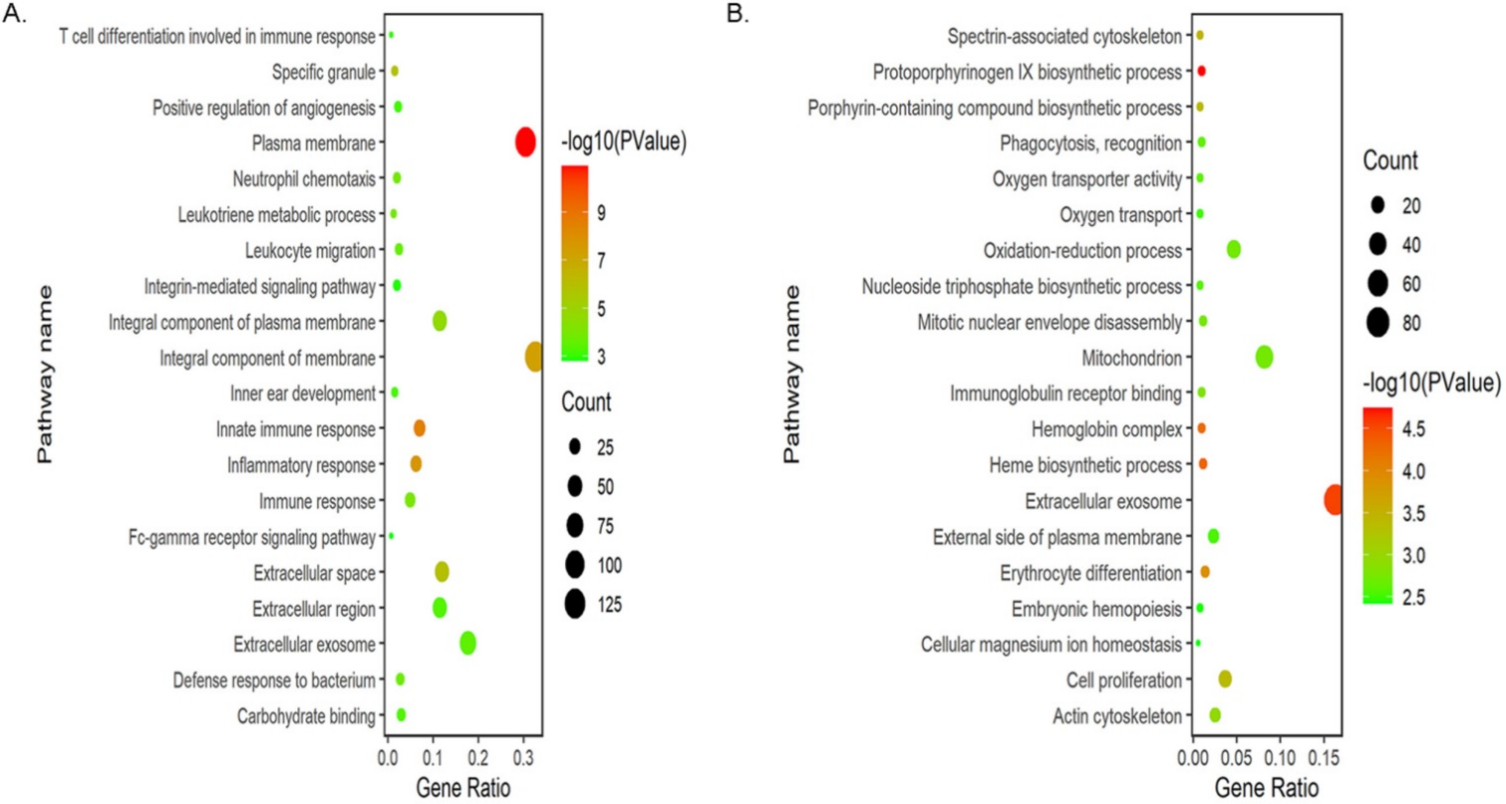
Bubble plots of GO enrichment analysis of DEGs. (**A**) Bubble plot of GO enrichment analysis of up-regulated DEGs. (**B**) Bubble plot of GO enrichment analysis of down-regulated DEGs.

To explore the functional roles of the DEGs, we performed GO enrichment analysis of up-regulated and down-regulated DEGs by using DAVID gene annotation tool. It turned out that obvious differences were enriched in BPs, MFs and CCs among the 796 DEGs. For BPs, the up-regulated DEGs were primarily enriched in immune response, including innate, adaptive immune response and T cell differentiation involved in immune response, suggesting that these DEGs could significantly associate with the immune system of myeloma LC/SP cells. Besides, these genes were also significantly enriched in inflammatory response, leukotriene metabolic process and neutrophil chemotaxis. The down-regulated DEGs were significantly enriched in protoporphyrinogen IX biosynthetic process, heme biosynthetic process and erythrocyte differentiation, indicating that the down-regulated DEGs may be relevant to the development and differentiation of erythrocytes. In the CCs group, the up-regulated DEGs were significantly involved in plasma membrane, integral component of membrane and extracellular space. In addition, down-regulated genes were largely enriched in the extracellular exosome and hemoglobin complex. Regarding MFs category, the up-regulated genes were mainly enriched in the binding of carbohydrate, calcium ion and arachidonic acid. Moreover, the most significantly enriched GO terms for down-regulated genes were immunoglobulin receptor binding, oxygen transporter activity and NAD activity. GO enrichment analysis results were displayed in Figure 3 and Table 2.

### KEGG pathway enrichment analysis of DEGs

**Figure 4:**
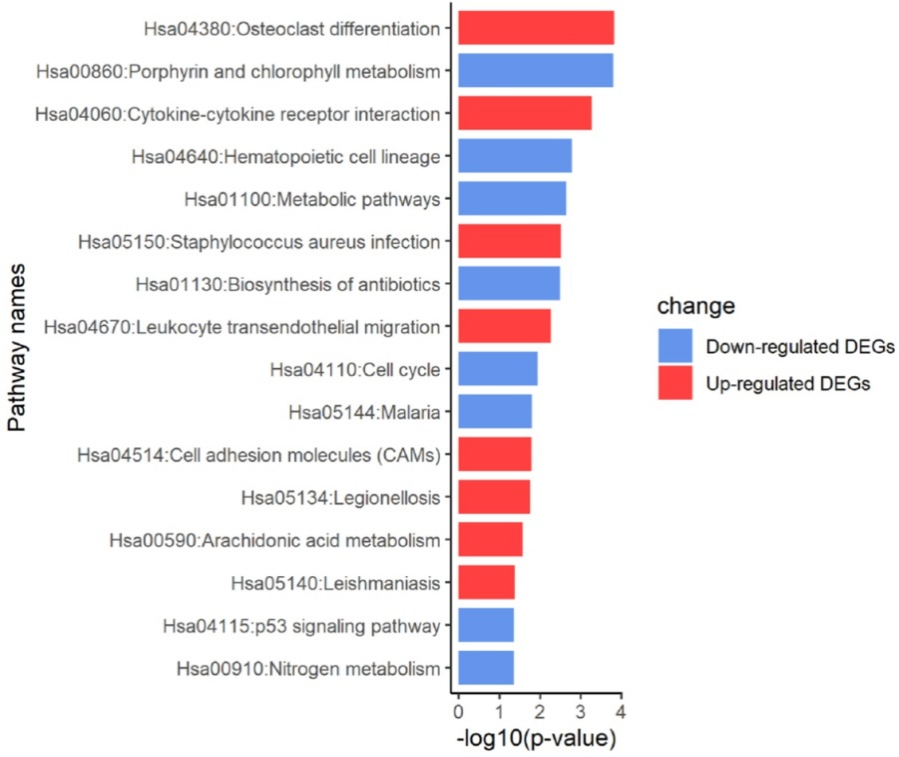
KEGG pathway analysis of DEGs.

**Figure 5:**
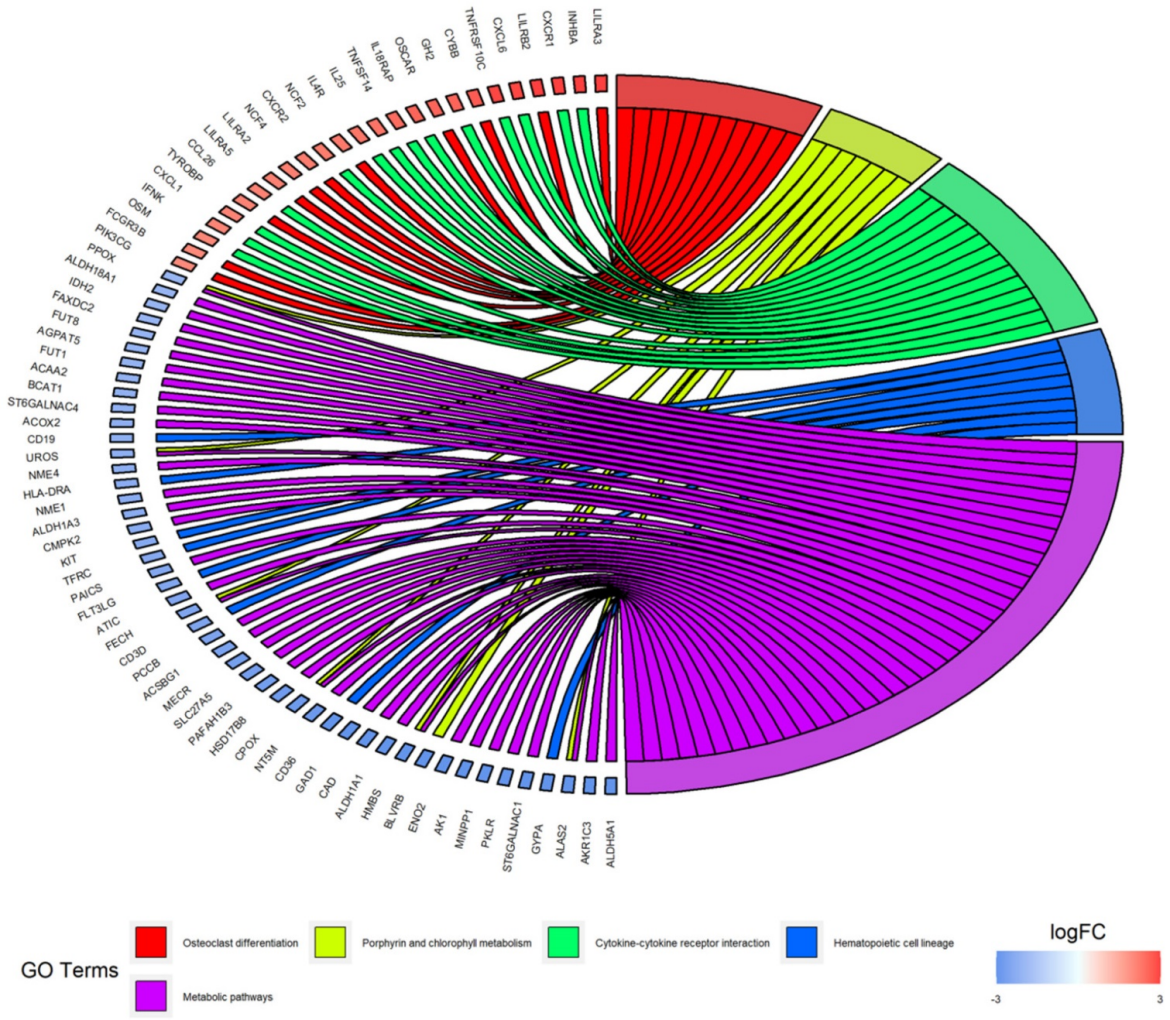
Distribution of DEGs in myeloma LC/SP cells for the top 5 KEGG enriched pathways.

According to the KEGG pathway enrichment analysis of up- and down-regulated DEGs, the up-regulated DEGs were mainly enriched in osteoclast differentiation, cytokine-cytokine receptor interaction, Staphylococcus aureus infection, leukocyte transendothelial migration and cell adhesion molecules. Furthermore, enrichment of down-regulated DEGs was mostly in the porphyrin and chlorophyll metabolism, hematopoietic cell lineage and metabolic pathways. KEGG analysis results were displayed in Figure 4 and Figure 5, and the detailed analysis results of the top 5 pathways were shown in Table 3.

### PPI network construction and modular analysis

**Figure 6:**
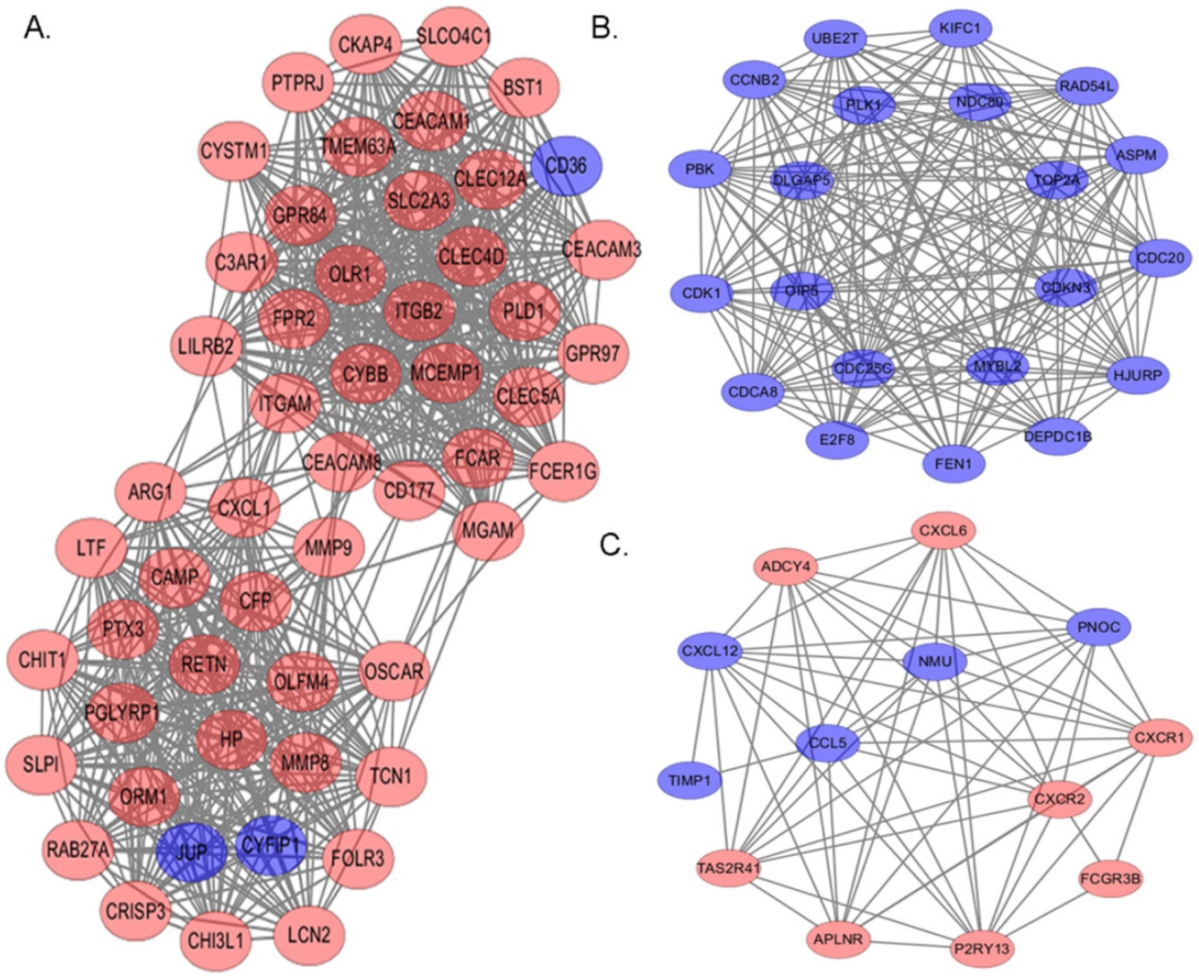
The top 3 modules with relatively high scores from the protein-protein interaction network. Red: up-regulation; Blue: down-regulation. (**A**) Module 1 with 53 nodes and 684 edges was significantly enriched in defense response, immune system process and immune response. (**B**) Module 2 with 21 nodes and 197 edges was significantly enriched in cell cycle phase, cell cycle and M phase of mitotic cell cycle. (**C**) Module 3 with 13 nodes and 60 edges was significantly enriched in G-protein coupled receptor protein, signaling pathway and chemotaxis.

On the base of STRING online database and Cytoscape software, we established a PPI network of these DEGs in myeloma LC/SP cells, with 610 nodes and 2922 edges identified, including 288 up-regulated and 322 down-regulated genes. Then, PPI module analysis was implemented by plug-ins MCODE in Cytoscape, and three significant modules were identified from the whole network. The top 3 modules with high scores were selected for display: module 1 contained 53 nodes and 684 edges (Figure 6A), module 2 contained 21 nodes and 197 edges (Figure 6B) and module 3 contained 13 nodes and 60 edges (Figure 6C). Subsequently, corresponding GO term enrichment analysis was performed by plug-ins BiNGO in Cytoscape. Genes in Module 1 were significantly enriched in the defense response, immune system process and immune response. Moreover, genes in Module 2 were mainly enriched in cell cycle phase, cell cycle and M phase of mitotic cell cycle. Additionally, genes in module 3 were primarily enriched in G-protein coupled receptor protein, signaling pathway and chemotaxis. The detailed information of the top 3 modules was shown in Table 4.

### Selection of hub genes from the PPI network

Among the previously described DEGs, significant hub genes were identified by plug-in cytoHubba of Cytoscape using MCC algorithm. The top 10 hub genes were ITGAM, MMP9, ITGB2, FPR2, C3AR1, CXCL1, CYBB, LILRB2, HP and FCER1G. The top 10 hub genes and their most relevant functions were displayed in Table 5.

### Survival analysis of DEGs

**Figure 7:**
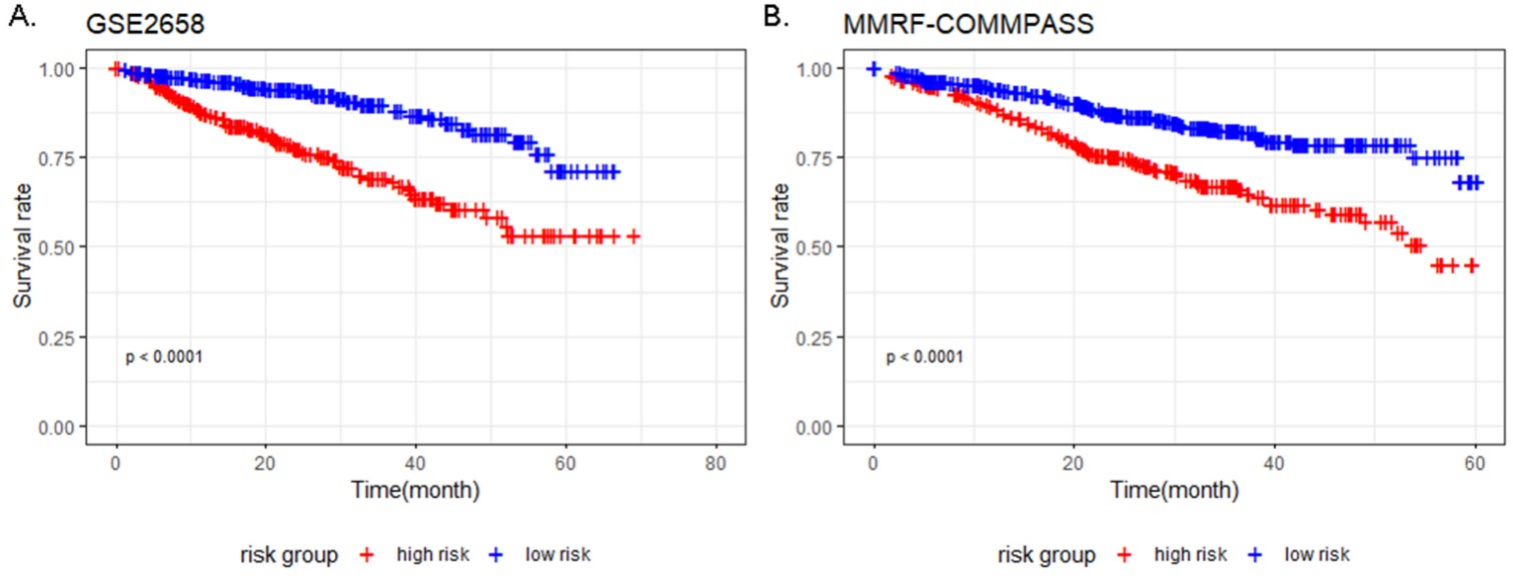
Kaplan-Meier survival analysis of 9 prognostic genes in MM patients in the training and validation datasets. (P-value < 0.0001 in both GSE2658 and MMRF-COMMPASS). (**A**) Kaplan-Meier survival analysis of 9 prognostic genes in MM patients in GSE2658. (**B**) Kaplan-Meier survival analysis of 9 prognostic genes in MM patients in MMRF-COMMPASS.

The result of univariate Cox analysis showed 76 survival related genes (Table S1; 19 up-regulated and 57 down-regulated) (*P*-value < 0.01). Afterwards, the 76 genes were fitted into the multivariate Cox proportional hazards analysis, and 9 genes including TFF3, EPDR1, MACROD1, ARHGEF12, AMMECR1, NFATC2, HES6, PLEK2 and SNCA were identified with *P*-value < 0.05 (Table 6). The prognostic models of training and validation dataset containing 9 genes were constructed by discriminating the low-risk group from the high-risk group based on the respective median risk score. Kaplan-Meier curve showed that high-risk group had worse survival compared to the low-risk group in both training and validation dataset (Figure 7).

## Discussion

In the present study, we performed a bioinformatics analysis to identify DEGs between myeloma LC/SP cells and MP CD138^+^ cells to explore the molecular characteristics of LC/SP cells. Based on the gene expression profile, we screened a total of 796 DEGs, including 403 up-regulated and 393 down-regulated genes. Subsequently, deeper exploration of these DEGs were performed by bioinformatics methods, including GO and KEGG pathway enrichment analysis, PPI network construction and modules analysis, selection of hub genes and survival analysis.

GO enrichment analysis demonstrated that the up-regulated DGEs were significantly enriched in innate immune response, inflammatory response, plasma membrane and integral component of membrane. Firstly, according to our enrichment analysis, up-regulated DEGs were most enriched in innate immune response. As reported by Grivennikov SI et al., components of innate immunity such as macrophages, and DCs can either induce anti-tumor immune responses or promote tumor growth and progression depending on their morphological and phenotypic subtypes 30. In multiple solid tumor models, the presence of tumor infiltrating macrophages (TAM) in tumor lesions can promote “stemness” property of cancer cells 31. However, for MM, the association between SP cells and innate immune response has not been explained clearly yet. With regard to up-regulated DEGs enriched in immune response, it is now well-established that FGR, CXCL1, NLRC4 and S100A9 influence the pathogenesis of cancer by modulating immune responses and promoting progression, aggressiveness and cell survival 32-35. Besides, up-regulated DGEs were also significantly enriched in inflammatory response. Then, the up-regulated DEGs were significantly involved in plasma membrane and integral component of membrane. It has been reported that the unique and specific makeup and arrangement of cell membranes of cancer cells are critical for cells to survive, grow and proliferate^36^. The enrichment analysis indicated that myeloma LC/SP cells may have unique plasma membrane and integral component of membrane compared to MP cells, and targeting the uniqueness may lead to the reduction of SP cells. Additionally, the down-regulated DEGs were significantly enriched in protoporphyrinogen IX and heme biosynthetic process, hemoglobin complex and erythrocyte differentiation, indicating that the down-regulated DEGs may be relevant to the development and differentiation of erythrocytes. Moreover, extracellular exosome was also enriched significantly. Exosomes are membranous structures that carry signaling molecules and regarded as important mediators of inter-cellular communication in health and disease 37. Studies have revealed a strong cross-talk between the MM cells and their microenvironment in the bone marrow, which leads to the final phenotype of a typical MM patient 38, 39. This result demonstrated that extracellular exosome may function significantly in this small fraction of MM cells.

KEGG pathway enrichment analysis showed some DEGs were significantly enriched in osteoclast differentiation, porphyrin and chlorophyll metabolism and cytokine-cytokine receptor interaction. Osteolytic bone disease is the hallmark of MM, which deteriorates the quality of life of myeloma patients. It has been demonstrated that increased osteoclast activity is one of the important mechanisms 40. Among the DEGs, some studies found that PIK3CG, LILRB2 and CYBB could regulate the differentiation of osteoclast, which highlighted the possible biological significance of LC/SP cells in osteoclast differentiation 41-43. ALAS2, significantly enriched in porphyrin and chlorophyll metabolism pathway, plays a key role in erythropoiesis by regulation of erythroid heme synthesis 44. As already described in GO analysis, parts of the down-regulated DEGs may associate with the erythropoiesis, indicating that myeloma LC/SP cells may impair the erythropoiesis. CXCL1, significantly enriched in cytokine-cytokine receptor interaction pathway, could result in the enhancement of MM cell viability and migration 45. Additionally, Staphylococcus aureus infection was enriched in KEGG pathway analysis. It's well known that infectious complications are a frequent cause of morbidity and mortality of MM 46. A prospective study observed the rate of infections varied in different phases of MM, and the most infections were clinically diagnosed as pneumonia and bronchopneumonia caused by Haemophilus influenzae or Streptococcus pneumonia in early-stage MM 47. As to Staphylococcus aureus infection, a recent study showed that Staphylococcus aureus bacteremia (SAB) may be an early prognostic indicator of cancer because of the phenomenon that patients with SAB were more likely to die from cancer than the general population 48. Furthermore, an association between SAB and risk of multiple myeloma was described 49. According to our results, it is presumable that myeloma SP cells may be relevant to infectious complications, especially the SAB infection, which opens a fundamental direction to understand infections for patients suffering from MM. However, the results need to be confirmed in further basic and clinical research. In brief, the enriched GO and KEGG pathways partly clarified the specific molecular characteristics of myeloma SP cells.

Then a PPI network of these DEGs in myeloma LC/SP cells was established, containing 610 nodes and 2922 edges. In the network, we selected three significant modules through the degree of importance and corresponding GO term enrichment analysis was performed, which indicated that myeloma LC/SP cells was significantly associated with immune system process, immune response and cell cycle, basically consistent with what we mentioned above. Subsequently, ten significant hub genes have been identified, containing ITGAM, MMP9, ITGB2, FPR2, C3AR1, CXCL1, CYBB, LILRB2, HP and FCER1G.

ITGAM was identified as the top 1 hub gene and had the highest degree of connectivity. ITGAM encodes CD11b, a component of the macrophage-1 antigen complex (Mac1, also known as complement receptor 3 [CR3]), which together with CD18, form Mac-1 or CR3, a protein that mediates leukocyte adhesion, migration, and phagocytosis in different cells 50-53. CD11b contributes to the phagocytosis of opsonized particles, including apoptotic cells and immune complex 53. What's more, CD11b is defined as a marker for myeloid-derived suppressor cells, which is reported to be harnessed by malignant cells to restrain antitumor immunity and promote malignant expansion or refractoriness to treatment 54-56. It has been considered as a poor prognostic factor in MM 57 and AML patients 58-61. But association between SP cells and CD11b remains unclear, and it is presumable that CD11b may participate in the regulation of biology of LC/SP cells and its up-regulation may promote expansion of MM. ITGB2 produces a protein, known as CD18, which is a cell surface marker expressed on lymphocytes 62 and is involved in cell adhesion and cell-surface mediated signaling 63. It has been demonstrated that mutation in the ITGB2 gene could lead to leukocyte adhesion deficiency 64. And its expression in CLL cells predicts disease progression 65. In MM cell line, ITGB2 is overexpressed in vincristine resistant cell line 66. Nonetheless, the correlation of drug resistance and ITGB2 requires further analysis.

As a key adhesion receptor, integrin CD11b/CD18 meditates leukocyte migration and immune functions 67. Recently, several studies have investigated that the adhesion and angiogenesis system is vital to propagate MM progression with a vicious cycle by the endothelial-MM interaction. β integrin has been described to participate in the homing and adhesion of endothelial progenitor cells to sites of vascular remodeling 68, 69. It has been uncovered that some integrins were detected in high levels in MM, while in non-detectable levels in non-active MM and MGUS patients, suggesting the adhesion molecules support the interactions between MM and the microvasculature and facilitate disease progression 70. Furthermore, junctional adhesion molecule A has been identified as a key mediator of MM progression by promoting MM-associated angiogenesis and an independent prognostic factor for both newly diagnosed MM and relapsed/refractory MM 71, 72. Similarly, our enrichment analysis of up-regulated DEGs had identified positive regulation of angiogenesis and cell adhesion, demonstrating that myeloma SP cells may be relevant to angiogenesis and cell adhesion to propagate MM progression.

The significantly up-regulated MMP9 gene (matrix metallopeptidase 9), one of the most widely investigated matrix metalloproteinases, is a significant protease which plays vital roles in many biological processes and cancer cell invasion, metastasis and angiogenesis 73. Recently, MMP9 has been identified as a potential biomarker for several cancers 74-78. As far as MM concerned, previous reports indicated that the expression of MMP9 in MM cells promote MM invasion 79-84, which may highlight the role of increased neovascularization in MM progression. In fact, it has been testified that angiogenesis, which is linked to aberrant expression of pro-angiogenic and down-regulation of anti-angiogenic genes 85, is a feature of MM progression through the transition from MGUS to MM, and plays a role in medullary and extramedullary dissemination 86, 87. Recently, several angiogenic factors in active MM have been discovered, like VEGF (Vascular endothelial growth factor), FGF-2 (Fibroblast growth factor-2), HGF (Hepatocyte growth factor), MMP-2/9 and so on 88. Additionally, data have shown that mTORC2 is involved in MM angiogenesis 89, and activation of the PI3K/AKT/mTOR pathway regulates pro-angiogenic factors of MMP-9 90. Besides, Notch signaling has been investigated in the cross talk between endothelial cells and MM cells to enable angiogenesis 91. Consistently, in our GO enrichment analysis, the up-regulated DEGs were enriched in positive regulation of angiogenesis, suggesting the role of angiogenesis of myeloma SP cells. In a conclusion, neovascularization and positive regulation of angiogenesis may be regarded as potential factors in modulating MM progression and deserving prognostic roles. Nevertheless, its biological mechanisms have not clearly revealed yet. In the future, additional studies are needed to further confirm the mechanisms of angiogenesis in myeloma SP cells.

Extramedullary disease of MM remains a key area of therapeutic challenge, and the expression of adhesion molecules and changes in angiogenesis concerning mostly VEGF, MMP-9 and others are involved in extramedullary spread of MM cells 92, 93. Plasma cells from extramedullary plasmacytomas showed angiogenesis related expression 94. What's more, neovascularization can promote the growth of plasmacytomas 95. These discoveries supported the idea that increased angiogenesis could facilitate malignant plasma cells growth outside the BM microenvironment. However, there are still unsolved questions on extramedullary myeloma involvement, especially on the relevant association with myeloma SP cells, which require further study.

In our survival analysis, to evaluate the association between the DEGs and clinical survival of MM patients and predict the prognosis of MM patients, we revealed 9 DEGs including TFF3, EPDR1, MACROD1, ARHGEF12, AMMECR1, NFATC2, HES6, PLEK2 and SNCA to be significantly associated with survival and established a survival prediction model based on the 9 genes. Stratified by risk score, a significantly different clinical outcome of MM patients were showed by the Kaplan-Meier curve in both training and validation datasets. However, further investigation of these genes in clinical research is warranted.

There are some limitations in our study. Firstly, the identification of DEGs profile was performed without external validation of other databases because of the absence of available data about SP cells compared to MP cells in MM. Second, we didn't evaluate the correlation of the prognostic model with clinicopathological characteristics. Thirdly, our study was only analyzed based on bioinformatics analysis. Hence, further investigations are warranted to validate the results and enhance our understanding of the biological role of these genes in MM.

## Conclusions

To sum up, we performed a comprehensive bioinformatics analysis on microarray data of myeloma LC/SP cells. DEGs were identified to be significantly enriched in various pathways, especially positive regulation of angiogenesis and cell adhesion. The results of this study increase our understanding of novel biomarkers of myeloma LC/SP cells, prediction of MM prognosis and potential therapeutic targets. Nevertheless, further relevant studies are needed to confirm the identified DEGs and pathways in LC/SP cells of MM.

## Supplementary Material

Supplementary table.

Supplementary raw data and code.

## Figures and Tables

**Table 1:** Detailed information on the top 10 DEGs in the analysis

Symbol	Name	AveExpr	*t*	*P*-Value	adj.*P*-Val	*B*	logFC
SLC27A5	solute carrier family 27 member 5	6.641	-9.704	7.23E-08	1.46E-03	7.969	-2.685
MARCHF11	membrane associated ring-CH-type finger 11	4.541	7.686	1.38E-06	1.16E-02	5.451	3.293
RBFA	ribosome binding factor A	6.812	-7.379	2.26E-06	1.16E-02	5.015	-3.401
GFI1B	growth factor independent 1B transcriptional repressor	6.468	-6.874	5.21E-06	1.16E-02	4.267	-4.616
ECI2	enoyl-CoA delta isomerase 2	9.317	-6.799	5.91E-06	1.16E-02	4.152	-3.099
SNRK-AS1	SNRK antisense RNA 1	7.699	6.755	6.38E-06	1.16E-02	4.084	2.448
PTK2	protein tyrosine kinase 2	6.761	-6.680	7.25E-06	1.16E-02	3.968	-3.842
RPS6KL1	ribosomal protein S6 kinase like 1	6.489	-6.673	7.34E-06	1.16E-02	3.957	-2.658
LRRC45	leucine rich repeat containing 45	6.612	-6.639	7.78E-06	1.16E-02	3.903	-2.562
KBTBD7	kelch repeat and BTB domain containing 7	10.589	6.635	7.83E-06	1.16E-02	3.898	2.122

**Table 2:** Gene Ontology term enrichment analysis of DEGs in LC/SP cells of MM.

Expression	Category	ID	Term	Count	PValue
	BP	GO:0045087	innate immune response	28	3.13E-09
	BP	GO:0006954	inflammatory response	25	2.07E-08
	BP	GO:0006955	immune response	20	8.02E-05
	BP	GO:0006691	leukotriene metabolic process	5	8.69E-05
	BP	GO:0030593	neutrophil chemotaxis	8	1.07E-04
	CC	GO:0005886	plasma membrane	122	1.23E-11
	CC	GO:0016021	integral component of membrane	131	5.17E-08
UP-DEGs	CC	GO:0005615	extracellular space	48	1.22E-06
	CC	GO:0042581	specific granule	6	1.47E-06
	CC	GO:0005887	integral component of plasma membrane	46	2.27E-05
	MF	GO:0030246	carbohydrate binding	12	4.29E-04
	MF	GO:0005509	calcium ion binding	24	1.91E-03
	MF	GO:0050544	arachidonic acid binding	3	2.63E-03
	MF	GO:0004198	calcium-dependent cysteine-type endopeptidase activity	4	4.76E-03
	MF	GO:0005536	glucose binding	3	1.36E-02
	BP	GO:0006782	protoporphyrinogen IX biosynthetic process	5	1.81E-05
	BP	GO:0006783	heme biosynthetic process	6	4.80E-05
	BP	GO:0030218	erythrocyte differentiation	7	1.31E-04
	BP	GO:0006779	porphyrin-containing compound biosynthetic process	4	4.09E-04
	BP	GO:0008283	cell proliferation	19	4.20E-04
	CC	GO:0070062	extracellular exosome	84	2.77E-05
	CC	GO:0005833	hemoglobin complex	5	5.86E-05
DOWN-DEGs	CC	GO:0014731	spectrin-associated cytoskeleton	4	3.66E-04
	CC	GO:0015629	actin cytoskeleton	13	1.08E-03
	CC	GO:0005739	mitochondrion	42	1.84E-03
	MF	GO:0034987	immunoglobulin receptor binding	5	1.51E-03
	MF	GO:0005344	oxygen transporter activity	4	2.28E-03
	MF	GO:0004029	aldehyde dehydrogenase (NAD) activity	4	4.07E-03
	MF	GO:0051015	actin filament binding	9	4.36E-03
	MF	GO:0004064	arylesterase activity	3	5.39E-03

**Table 3:** KEGG pathway enrichment analysis of DEGs in LC/SP cells of MM

Expression	Category	Term	Count	*P*-Value
	hsa04380	Osteoclast differentiation	11	1.50E-04
	hsa04060	Cytokine-cytokine receptor interaction	14	5.51E-04
UP-DEGs	hsa05150	Staphylococcus aureus infection	6	3.15E-03
	hsa04670	Leukocyte transendothelial migration	8	5.44E-03
	hsa04514	Cell adhesion molecules (CAMs)	8	1.64E-02
	hsa00860	Porphyrin and chlorophyll metabolism	7	1.63E-04
	hsa04640	Hematopoietic cell lineage	8	1.65E-03
DOWN-DEGs	hsa01100	Metabolic pathways	39	2.24E-03
	hsa01130	Biosynthesis of antibiotics	12	3.24E-03
	hsa04110	Cell cycle	8	1.16E-02

**Table 4:** The top 5 significantly enriched GO terms and corresponding gene information in module analysis

Modules	GO-ID	*P*-value	corr *P*-value	*x*	Description	Genes in test set
	6952	2.31E-16	2.51E-13	20	defense response	ORM1|CRISP3|ITGB2|HP|CYBB|RAB27A|CXCL1|FPR2|LILRB2|PLD1|CFP|CLEC4D|CLEC5A|C3AR1|LCN2|OLR1|PTX3|PGLYRP1|CAMP|LTF
	2376	6.99E-13	3.80E-10	20	immune system process	ITGAM|ARG1|CRISP3|ITGB2|CYBB|RAB27A|CXCL1|LILRB2|CFP|MMP9|FCAR|CHIT1|BST1|CLEC4D|C3AR1|LCN2|PTX3|CEACAM8|PGLYRP1|LTF
module 1	6955	1.24E-11	4.49E-09	16	immune response	ARG1|CRISP3|CYBB|RAB27A|CXCL1|LILRB2|CFP|FCAR|CHIT1|BST1|CLEC4D|LCN2|PTX3|CEACAM8|PGLYRP1|LTF
	5576	3.52E-10	9.56E-08	24	extracellular region	ORM1|ARG1|CRISP3|HP|CXCL1|RETN|OLFM4|MMP8|CFP|MMP9|FCAR|CHIT1|TCN1|CEACAM1|SLPI|LCN2|OLR1|CHI3L1|PTX3|CEACAM8|FOLR3|PGLYRP1|CAMP|LTF
	30246	6.48E-09	1.38E-06	11	carbohydrate binding	CHIT1|ITGAM|CLEC4D|CLEC12A|ARG1|CLEC5A|OLR1|CHI3L1|PTX3|PGLYRP1|LTF
	22403	2.21E-18	1.13E-15	14	cell cycle phase	PLK1|CDCA8|CDC25C|NDC80|CDC20|CCNB2|ASPM|KIFC1|CDK1|PBK|RAD54L|OIP5|DLGAP5|CDKN3
	7049	3.54E-18	1.13E-15	16	cell cycle	PLK1|HJURP|CDCA8|CDC25C|NDC80|CDC20|CCNB2|ASPM|KIFC1|CDK1|PBK|RAD54L|OIP5|DLGAP5|CDKN3|E2F8
module 2	87	6.92E-18	1.48E-15	12	M phase of mitotic cell cycle	CDC20|CCNB2|ASPM|KIFC1|PLK1|CDK1|PBK|CDCA8|OIP5|CDC25C|NDC80|DLGAP5
	279	9.72E-18	1.56E-15	13	M phase	PLK1|CDCA8|CDC25C|NDC80|CDC20|CCNB2|ASPM|KIFC1|CDK1|PBK|RAD54L|OIP5|DLGAP5
	278	2.74E-17	3.51E-15	13	mitotic cell cycle	PLK1|CDCA8|CDC25C|NDC80|CDC20|CCNB2|ASPM|KIFC1|CDK1|PBK|OIP5|DLGAP5|CDKN3
	7186	1.94E-08	8.23E-06	7	G-protein coupled receptor protein signaling pathway	CXCL12|CXCR1|CXCR2|PNOC|NMU|ADCY4|APLNR
	42330	8.82E-08	1.25E-05	5	taxis	CXCL6|CXCL12|CXCR1|CCL5|CXCR2
module 3	6935	8.82E-08	1.25E-05	5	chemotaxis	CXCL6|CXCL12|CXCR1|CCL5|CXCR2
	7610	4.59E-07	4.18E-05	6	behavior	CXCL6|CXCL12|CXCR1|CCL5|CXCR2|NMU
	4918	4.93E-07	4.18E-05	2	interleukin-8 receptor activity	CXCR1|CXCR2

**Table 5:** The top 10 hub genes and their most relevant functions

Symbol	Gene name	Degree	Relevant function	Reference
ITGAM	integrin subunit alpha M	78	A poor prognostic factor in MM and AML patients;	57-61
MMP9	Matrix metallopeptidase 9	64	Participates in the breakdown of extracellular matrix; Promotes invasion of MM;	73, 79-84
ITGB2	Integrin subunit beta 2	62	Involved in cell adhesion and cell-surface mediated signaling; Associated with drug resistance to chemotherapy in MM cell line;	63, 66
FPR2	Formyl-peptide receptor-2	52	Associated with invasion and metastasis of some cancers;	96-98
C3AR1	complement C3a receptor 1	51	Involved in drug resistance to chemotherapy in AML cell; Predicts overall survival of AML;	99
CXCL1	C-X-C motif chemokine ligand 1	51	Associated with the growth and progression of some cancers;	100-102
CYBB	cytochrome b-245 beta chain	48	Involved in the progression of some cancers by promotion of angiogenesis;	103, 104
LILRB2	leukocyte immunoglobulin like receptor B2	46	Inhibits stimulation of an immune response; Promotes tumor progression;	105-107
HP	haptoglobin	44	NA	-
FCER1G	Fc fragment of IgE receptor Ig	43	Associated with disease progression in lymphoma and some solid cancers; Deficient expression represents T-cell immunodeficiency in CLL.	108-110

**Table 6:** Multivariate Cox regression analysis of 9 genes used for constructing the prognostic model

Symbol	Coefficient	HR	Lower 95%CI	Upper 95%CI	*P*-value
TFF3	0.566	1.762	1.240	2.503	0.002
EPDR1	0.492	1.636	1.132	2.364	0.009
MACROD1	0.745	2.107	1.189	3.734	0.011
ARHGEF12	-0.540	0.583	0.389	0.874	0.009
AMMECR1	0.862	2.369	1.142	4.913	0.020
NFATC2	0.679	1.972	1.258	3.090	0.003
HES6	0.513	1.671	1.091	2.560	0.018
PLEK2	0.527	1.694	1.041	2.758	0.034
SNCA	0.759	2.135	1.216	3.749	0.008
