# Supplementary material for: Identification of Key Genes and Pathways in Myeloma side population cells by Bioinformatics Analysis
Source: Int J Med Sci. 2020 Jul 25;17(14):2063–76. doi: 10.7150/ijms.48244 (PMC7484674; doi:10.7150/ijms.48244)
Supplement: Supplementary file 1 — Supplementary table. [file ijmsv17p2063s1.pdf]

Supplementary Table S1:

Univariate Cox regression analysis of the DEGs associated with disease specific survival (DSS) of MM.

DEGs	HR	Lower 95%CI	Upper 95%CI	Cox *P*-value
**Upregulated (n=19)**				
KIF21B	1.37	1.18	1.59	0.000037
TFF3	1.52	1.22	1.90	0.000203
TYROBP	0.59	0.44	0.79	0.000400
CCPG1	0.50	0.32	0.77	0.001494
GH2	0.54	0.36	0.80	0.002079
DSCR8	1.26	1.09	1.45	0.002259
TMEFF2	0.73	0.59	0.90	0.002779
LILRA3	0.46	0.28	0.77	0.002933
LIN7A	0.66	0.50	0.87	0.003299
TNNI2	0.56	0.38	0.83	0.004072
CABP7	0.74	0.60	0.91	0.004505
GSN	0.69	0.53	0.89	0.004844
LPGAT1	1.58	1.13	2.21	0.006998
LTA4H	0.52	0.32	0.84	0.007062
CNN3	0.85	0.76	0.95	0.004928
ORM1	0.74	0.59	0.93	0.008104
TNFSF14	0.62	0.43	0.89	0.009181
CKAP4	0.62	0.44	0.89	0.009583
PRAM1	0.57	0.37	0.87	0.009837
**Downregulated (n=57)**				
RBFA	2.25	1.60	3.15	0.000003
DEPDC1B	1.72	1.37	2.17	0.000004
UBE2T	2.00	1.48	2.69	0.000005
EPDR1	1.67	1.32	2.10	0.000017
CDK1	1.88	1.41	2.51	0.000019
E2F8	1.88	1.40	2.52	0.000026
HMGN5	1.62	1.28	2.04	0.00005
ATIC	3.46	1.90	6.30	0.000051
TOP2A	1.40	1.19	1.65	0.000052
CDKN3	1.59	1.26	2.02	0.000120
CCNB2	1.54	1.23	1.93	0.000133
KIFC1	1.54	1.23	1.92	0.000137
PPOX	2.88	1.67	4.99	0.000154
DLGAP5	1.86	1.34	2.58	0.000203
ASPM	1.42	1.18	1.71	0.000208
HJURP	1.55	1.23	1.95	0.000223
CRIP1	1.22	1.10	1.35	0.000246

MACROD1	0.53	0.37	0.74	0.000251
NAP1L5	0.70	0.57	0.85	0.000258
UROS	0.51	0.36	0.73	0.000274
TAL1	0.56	0.40	0.76	0.000296
OIP5	1.63	1.25	2.12	0.000324
MYBL2	1.35	1.14	1.60	0.000534
MEST	1.53	1.19	1.98	0.001114
C1orf112	1.38	1.14	1.69	0.001288
C12orf75	1.34	1.12	1.16	0.001486
PAICS	2.29	1.37	3.82	0.001489
FECH	1.48	1.16	1.88	0.001509
PNOC	0.57	0.40	0.81	0.001979
CDC20	1.46	1.14	1.86	0.002229
HMCN2	0.50	0.31	0.79	0.003244
ARHGEF12	0.71	0.56	0.89	0.003883
RPP40	1.96	1.24	3.08	0.003910
CA2	1.31	1.09	1.57	0.003987
CYB5A	2.15	1.28	3.62	0.004009
CHEK2	1.49	1.13	1.96	0.004887
AMMECR1	2.04	1.24	3.35	0.004953
PBK	1.47	1.12	1.92	0.005116
CDC25C	1.62	1.15	2.27	0.005253
ATP8B2	1.73	1.18	2.54	0.005388
HLA-DPA1	0.78	0.66	0.93	0.005411
HBZ	0.67	0.50	0.89	0.005437
HLA-DPB1	0.76	0.63	0.92	0.005540
NDC80	1.36	1.09	1.69	0.005697
BIN1	0.75	0.61	0.92	0.006124
FKBP1B	0.75	0.61	0.93	0.007393
NFATC2	1.46	1.11	1.93	0.007542
BOLA3	2.01	1.20	3.35	0.007712
HES6	0.69	0.53	0.91	0.007919
NUP210	1.83	1.17	2.86	0.008541
PLEK2	1.50	1.11	2.03	0.008664
SLC41A1	2.27	1.23	4.20	0.008865
SNCA	1.62	1.13	2.34	0.009082
BTN3A3	0.64	0.46	0.90	0.009406
NMU	1.21	1.05	1.40	0.009806
CDC42BPA	1.34	1.07	1.68	0.009852
GAD1	0.65	0.46	0.90	0.009995
